# Application of Furcellaran Nanocomposite Film as Packaging of Cheese

**DOI:** 10.3390/polym13091428

**Published:** 2021-04-28

**Authors:** Agnieszka Pluta-Kubica, Ewelina Jamróz, Gohar Khachatryan, Adam Florkiewicz, Pavel Kopel

**Affiliations:** 1 Department of Animal Product Processing, Faculty of Food Technology, University of Agriculture in Krakow, Balicka 122, PL-30-149 Krakow, Poland; agnieszka.pluta-kubica@urk.edu.pl; 2 Department of Chemistry, Faculty of Food Technology, University of Agriculture in Krakow, Balicka 122, PL-30-149 Krakow, Poland; ewelina.jamroz@urk.edu.pl (E.J.); gohar.khachatryan@urk.edu.pl (G.K.); 3 Department of Food Analysis and Qu4ality Assessment, Faculty of Food Technology, University of Agriculture in Krakow, Balicka 122, PL-30-149 Krakow, Poland; adam.florkiewicz@urk.edu.pl; 4 Department of Inorganic Chemistry, Faculty of Science, Palacky University, 17. listopadu 12, CZ-771 46 Olomouc, Czech Republic

**Keywords:** active packaging, nanofiller, gouda, quark, shelf life

## Abstract

There is a serious need to develop and test new biodegradable packaging which could at least partially replace petroleum-based materials. Therefore, the objective of this work was to examine the influence of the recently developed furcellaran nanocomposite film with silver nanoparticles (obtained by an in situ method) on the quality properties of two cheese varieties: a rennet-curd (gouda) and an acid-curd (quark) cheese. The water content, physicochemical properties, microbiological and organoleptic quality of cheese, and migration of silver nanoparticles were examined. Both the number of *Lactococcus* and total bacteria count did not differ during storage of gouda regardless of the packaging applied. The number of *Lactococcus* decreased in analogous quark samples. The use of the film slowed down and inhibited the growth of yeast in gouda and quark, respectively. An inhibitory effect of this film on mold count was also observed; however, only regarding gouda. The level of silver migration was found to be lower in quark than in gouda. The film improved the microbiological quality of cheeses during storage. Consequently, it is worth continuing research for the improvement of this film in order to enable its use in everyday life.

## 1. Introduction

Since the invention of plastic, more and more plastic packaging has been used in the food industry. Petroleum-based materials, when compared with others (e.g., paper, metal, or glass), have more favorable physical–mechanic properties, such as mechanical resistance, weight, and flexibility [1]. However, the application of non-biodegradable plastics as packaging materials represents a serious environmental problem worldwide [2]. Therefore, there is a serious need to develop and test new biodegradable packaging, which could at least partially replace petroleum-based materials.

Biopolymer-based materials account for a small percentage of the current global packaging market. Although their production costs are high and do not yet deliver economic benefits, the demand for these products is projected to increase rapidly over the coming decades and they will be widely used in packaging applications. Biopolymers meet the environmental requirements, but also exhibit some functional property limitations. However, biopolymers have already found applications in pharmacy and medicine, where cost is not as important as the function itself. Legal regulations on the part of the EU make it necessary to change the environmental awareness in the packaging industry [3].

Furcellaran (FUR) is a negatively charged sulphated polysaccharide, which is obtained from *Furcellaria lumbricallis*. It can interact with proteins and polysaccharides to create two-component and ternary films [4]. Thanks to its properties, it is an excellent film-forming matrix for active packaging materials [5]. Biopolymer films, in which FUR was one of the components, were used as packaging materials for food products such as: cheese [6], mini kiwi [7], linseed oil [8], salmon sushi [9], and fish [10,11].

Currently, one of the most efficient trends in the development of packaging materials is the application of nanotechnology [8]. Common nanocomposites incorporated in films as antimicrobial agents used for food packaging are based on metallic silver [12,13]. This component is highly toxic to a wide range of microorganisms, both as metallic and ionic silver. Moreover, it is characterized by low volatility and high temperature stability. Silver nanoparticles exhibit a high antimicrobial effect due to their ability to insert within the cell membrane and to release silver cations through oxidation [13]. Unfortunately, silver nanoparticles used in commercial packaging materials have recently been shown to migrate to food simulants [14], as well as from plasticized polyvinyl chloride (PVC) nanocomposites to food [15]. According to the Commission Regulation (EU) no. 10/2011, the maximum permitted amount of non-volatile compounds released from an article or a material into food simulants equals 60 mg/kg of food [15]. However, the migration limit for silver has not been established. Nevertheless, silver nanoparticles produced in the biopolymer solution are non-toxic and safe [16].

Gouda is a ripened semi hard to hard Dutch-type cheese made of cow milk. Nowadays it is one of the most popular cheeses in the world. Gouda is manufactured from raw or pasteurized milk (72 °C/15 s) using mesophilic starter cultures of lactic acid bacteria (LAB) and rennet [17,18]. It is brined before ripening and lasts 1–20 months [19].

Quark, which can be also called quarg, tvarog, or tvorog, is a type of un-ripened, soft, acid-curd cheese. Quark is manufactured by acid coagulation of cow milk due to acidification conducted by mesophilic starter cultures of LAB [20]. This cheese variety may be produced using a small quantity of rennet or without it. Acidification of inoculated milk is performed at 20–23 °C and lasts from 14 to 18 h until a pH of 4.6–4.8 is reached. If rennet is not added during manufacturing, a lower pH is essential to prepare quark with the same firmness [21]. Quark is characterized by a limited shelf life. As a result, it requires controlled refrigerated conditions during storage. The reason for this cheese’s short shelf life is its chemical composition, which constitutes a perfect medium for microorganism growth [22].

Gouda is produced and distributed with a dry rind, which may be coated, e.g., with paraffin-based ingredients. It can also be manufactured without formation of rind when ripening film is used, e.g., made of polythene [17,23,24]. Quark may be wrapped in parchment paper but the use of such packaging results in a very short shelf life (2–4 days). Therefore, this cheese variety is usually packed in plastic containers using a vacuum and a modified atmosphere which, compared to traditional wrapping in parchment paper, ensures a five to eight times longer shelf life [25]. Nevertheless, plastic packaging is most commonly used for both gouda and quark available in retail grocery stores.

Recently, Jamróz et al. [26] prepared and analyzed furcellaran nanocomposite films with graphene oxide (GO), multi-walled carbon nanotubes (MWCNTs), and silver nanoparticles (AgNPs). They concluded that only FUR + AgNPs exhibited an in vitro antimicrobial effect which correlated positively with the concentration of nanoparticles. Moreover, it was characterized by the lowest water solubility. However, it also exhibited high water vapor transmission rate values and was not colorless. This may limit its use as food packaging. Therefore, we hypothesize that the furcellaran film with silver nanoparticles may improve the microbiological quality of cheese; however, it may have a negative influence on their organoleptic features. Thus, the objective of this work was to examine the influence of the recently developed FUR + AgNPs film on quality properties of two different cheese varieties: a rennet-curd (gouda) and an acid-curd (quark) cheese. Water content, physicochemical properties, microbiological, and organoleptic quality were determined before and after storage. The degree of silver nanoparticle migration to the cheeses was also tested in order to assess whether it poses a threat to human health. To our knowledge, the influence of an active biodegradable film enriched with nanofillers such as AgNPs on a rennet-curd and an acid-curd cheese during storage presented in this work was investigated for the first time. Cheeses obtained by different coagulation methods were chosen because, according to our experience so far, a given type of foil may have a different impact on the quality properties of cheeses belonging to different types, e.g., soft rennet-curd cheese [6] and acid-curd cheese [27].

## 2. Materials and Methods

### 2.1. Materials

FUR (powdered EastGel type 7000) with Mw 2.951 × 10^5^ was obtained from Est-Agar AS (Karla village, Estonia). It contained carbohydrates (79.61%), protein (1.18%) and fat (0.24%). Silver nitrate, xylose, and glycerol were manufactured by Sigma-Aldrich (St. Louis, MO, USA). Linear low-density polyethylene (LLDPE) film was bought in a grocery store.

Samples of gouda (fat 26.0%, carbohydrates 0.0%, protein 25.0%, salt 1.6%) were manufactured from pasteurized cow milk by SM “MLEKPOL” (Grajewo, Poland). Samples of quark (fat 4.0%, carbohydrates 3.5%, protein 17.0%, salt 0.11%) were manufactured from pasteurized cow milk without rennet addition by OSM “RADOMSKO” (Nowy Targ, Poland). Samples were purchased in two independent series (all with different batch numbers) from a local market (Krakow, Poland) and transported in refrigerated conditions to the laboratory. The samples of each cheese variety were divided into two groups: wrapped in LLDPE film (control) and in FUR + AgNPs film (active). Every cuboid-shaped cheese piece weighed 150 ± 5 g. The samples of quark and gouda were stored for 2 weeks at 4 ± 1 °C and for 4 weeks at 8 ± 1 °C, respectively.

### 2.2. Preparation of an Active Film

The method of preparing FUR + AgNPs film was identical to the one previously described by Jamróz et al. [26] The films were prepared in our laboratory. Briefly, furcellaran (0.5 g) was dissolved in distilled water (50 mL). Then an aqueous solution of 0.1 M AgNO_3_ (0.2 mL) was added and stirred at 70 °C on a magnetic stirrer for 30 min. Xylose (2 mL) was added as a reducing agent to the film-forming solution and stirred overnight. When the color of the solution turned slightly brownish, a plasticizer—glycerol (0.5% *v/w* based on the solution)—was added. The prepared solution was poured onto a petri dish (diameter 14 cm) and dried in a hood for 48 h.

FUR + AgNPs film was characterized in detail in the previous work [26]. The AgNPs were observed by back-scattered electron imaging. They were homogenously distributed in the film and their size was 5–20 nm. Moreover, the formation of nanoparticles in the film-forming solution was confirmed using UV-vis absorption spectroscopy. Thickness, water content, solubility, swelling degree, water vapor transition rate, tensile strength, and elongation at break of the FUR + AgNPs film were as follows: 0.09 ± 0.0 mm, 13.72 ± 1.16%, 24.72 ± 2.27%, 386.44 ± 36.40%, 555.30 ± 1.8 g/m^2^/day, 25.46 ± 0.59%, and 26.59 ± 1.29%, respectively. The film was transparent. Its thermal properties were as follows: the peak temperature equaled 189.8 °C and the enthalpy was −119.23 J/g [26].

### 2.3. Water Content and Physicochemical Properties of Cheese

Water content, water activity, and pH were determined in the cheese samples directly after purchase and after storage. The content of water in gouda and quark was determined according to AOAC [28]. The water activity in cheese samples was measured using LabMaster-aw (Novasina AG, Lachen, Switzerland). The pH of gouda and quark was determined electrometrically using a pH-meter (CP-411, Elmetron, Zabrze, Poland). The water activity and pH of samples was measured according to Berti et al. [29] All aforementioned analyses were performed in triplicate.

### 2.4. Microbiological Quality of Cheese

*Lactococcus* count, total counts of bacteria (TBC), yeast, molds, and coliform count were determined directly after purchase and after storage. Test samples, initial suspension, and decimal dilutions of gouda and quark were prepared according to PN-EN ISO 6887-5:2010 [30]. Buffered peptone water (BioCorp, Warsaw, Poland) was used for suspension and dilutions and 10 g of each previously grated sample was weighed to a blender bag with lateral filter (BBAG-03, sterile, 400 mL, 190 mm × 300 mm, Corning Gosselin, Borre, France). Afterwards, 90 mL buffered peptone water was added. The homogenization lasted 3 min and was performed using a Stomacher device (Star Blender LB400, VWR, Radnor, PA, USA). Next appropriate decimal dilutions were prepared. Media such as M17, PCA, DRBC, and VRBL were purchased from BioCorp (Warsaw, Poland). The plates were incubated under aerobic conditions at 30 °C for 72 h (*Lactococcus* and TBC), at 25 °C for 120 h (yeast and molds) and at 30 °C for 24 h (coliforms). Reference methods were as follows: *Lactococcus* count: Ong and Shah [31]; TBC count: PN-EN ISO 4833-1:2013-12 [32]; yeast and mold count: PN-ISO 21527-1:2009 [33]; and coliform bacteria count: PN-ISO 4832:2007 [34]. Yeast and mold colonies were differentiated based on morphological differences [33].

### 2.5. Organoleptic Quality of Cheese

The organoleptic quality of gouda and quark samples was evaluated according to Baryłko-Pikielna and Matuszewska [35]. It was determined directly after wrapping at the beginning of storage and after storage. A 9-point hedonic scale (1–9 points referred to: extremely undesirable (1), very undesirable (2), undesirable (3), slightly undesirable (4), neither undesirable nor desirable (5), slightly desirable (6), desirable (7), very desirable (8), extremely desirable (9)) was applied. The following quality properties were evaluated: appearance in the packaging material, appearance after removing the packaging material, smell, consistency, and overall quality. Taste was not considered during the evaluation because adequate toxicological data of active packaging materials containing AgNPs are not yet available [15]. Every sample of gouda and quark was evaluated by 16 trained panelists.

### 2.6. Migration of Silver Nanoparticles

The silver content in gouda and quark samples was determined in triplicate by a validated Atomic Absorption Spectrometry method with electrothermal atomization (ETAAS) (Varian AA240Z, Varian Inc., Mulgrave, VIC, Australia) according to the procedure developed on the basis of PN-EN 14084:2004 [36]. The samples were analyzed directly after purchase and after storage in the active film. The cheeses after storage were analyzed as the rind and the interior of the cheese. The rind was the outer fragment of the sample, 1 mm thick, which was in contact with the packaging material. The interior was the part of the sample that was left after removing the rind. The rind and the interior were grated separately before the silver content analysis. The wet mineralization was conducted with the pressure microwave method (MarsXPress, CEM Corporation, Matthews, NC, USA), with nitric acid (Suprapur, MERCK, Darmstadt, Germany) and 10 mL of nitric acid was given for each 0.5 g of sample. The process of mineralization was conducted in 50 mL Teflon containers at maximum temperature of 200 °C. Certified Reference Materials NCS ZC73009 were used (China National Analysis Center for Iron and Steel) for checking the research method. This method has been fully validated and is subjected to the internal quality control procedure, according to PN-EN 13804:2013-06 [37]. The limit of quantification was 0.009 mg/kg.

### 2.7. Statistical Analysis

The obtained results were statistically analyzed using Statistica version 13.3 (TIBCO Software Inc., Palo Alto, CA, USA). Means and standard deviations were calculated. A one-way ANOVA was employed and the significance of differences between the means was established using the Tukey’s test. For the variables for which the assumptions of the analysis of variance were not met (on the basis of the Shapiro–Wilk and Levene’s test results), the Box–Cox transformation was used. When this was not successful, the nonparametric one-way ANOVA (Kruskal–Wallis test) and the multiple comparisons on ranks of several independent samples were performed. Student’s t-test was used for the variables for which the values were present in two groups. The results of organoleptic evaluation were statistically analyzed using the Kruskal–Wallis test by ranks.

## 3. Results and Discussion

An attempt was made to use the recently developed nanocomposite furcellaran film with silver nanoparticles obtained by an in situ method [26] as a packaging material for cheeses: a rennet-curd (gouda) and an acid-curd (quark) cheese. Nanocomposite films with furcellaran obtained by this method showed better performance properties than nanocomposite films obtained by an ex situ method [4]. Moreover, according to Jamróz et al. [26], the antimicrobial effect correlated positively with the concentration of nanoparticles. Therefore, the film with the highest content of AgNO_3_(aq) was chosen to be tested in our study (0.2 mL). Furthermore, FUR + 0.2 AgNPs was characterized by the greatest thickness as well as the lowest water solubility and swelling degree [26]. Determining the impact of the newly obtained packaging on a specific food product is one of the most important elements of the research procedure [38].

### 3.1. Water Content and Physicochemical Properties of Cheese

The content of water, water activity, and pH determined before wrapping and after storage of gouda and quark are presented in Table 1 and Table 2, respectively. The content of water and water activity did not differ significantly during storage in control whereas it decreased (*p* ≤ 0.05) in the samples wrapped in the active film, regardless of the type of cheese. Regarding the pH of gouda, it was not affected by storage time in control and decreased (*p* ≤ 0.05) in the cheese packed in the FUR + AgNPs film. On the other hand, pH of quark did not change during storage regardless of the packaging applied.

A similar decrease in water content was determined by Berti et al. [29] in gouda in edible coating containing natamycin and nisin as well as by Youssef et al. [39] in Ras cheese coated with chitosan/polyvinyl alcohol/glycerol suspension. The significant decrease in water content during the storage of both cheese varieties wrapped in the active film was caused by a much higher water vapor transmission rate (WVTR) of FUR + AgNPs film [26] than LLDPE [40]. Water activity decreased in the aforementioned samples, most likely as a consequence of decreased moisture. A similar reduction in water activity as well as moisture losses during storage were observed by Dmytrów et al. [22] in samples of quark, which was packed using MAP in polylactic acid (PLA) film. The authors concluded that these changes were caused by high WVTR of the packaging material.

Water activity can also be affected by changes in salt-in-moisture [29]. The salt-in-moisture of our examined cheeses probably increased during storage as the water content decreased. This may have occurred especially in gouda, which, unlike quark, is salted during production. The decrease in water activity during the storage of the control gouda could have been caused by proteolytic changes as free amino acids characterized by side chains with polar groups can interact with water molecules. This results in a decrease in water activity [41].

Acidity determined in gouda was typical for this cheese variety. According to Jo et al. [19], its pH ranges from 5.03 to 5.77. Contrary to our study, the pH of gouda in edible coating was found to be constant throughout the ripening for 30 days by Berti et al. [29] On the other hand, Jasińska et al. [25] determined a similar pH of quark packed in 50 μm PE film before and after 14 days of storage, which was in agreement with our findings. In addition, Dmytrów et al. [22] obtained the same observations regarding quarks packed in plastic and PLA film.

### 3.2. Microbiological Quality of Cheese

The results of microbiological analyses of gouda and quark are presented in Table 1 and Table 2, respectively. Unfortunately, it is not possible to draw conclusions about the effect of LLDPE nor the FUR + AgNPs film on the number of coliform bacteria in gouda and quark because coliforms were neither detected in fresh nor in stored samples.

Both the number of *Lactococcus* and TBC did not differ during the storage of gouda regardless of the packaging used. However, the number of *Lactococcus* decreased (*p* ≤ 0.05) in quark wrapped in LLDPE as well as in FUR + AgNPs film during storage. Moreover, the number of these microorganisms did not significantly differ between quark samples after storage was packed in different films. TBC was not affected during storage of quark regardless of the packaging applied. Therefore, the active film had the same effect on TBC and *Lactococcus* bacteria as LLDPE during storage of both cheese varieties. Different results were reported by Amjadi et al. [42], who investigated the influence of films containing zinc oxide nanoparticles on TBC in white cheese. They reported the inhibition of bacteria after nine days of storage.

*Lactococcus* was the dominant genus of bacteria enumerated in the examined cheeses as it is part of a typical mesophilic mixed strain starter culture used in the manufacturing of gouda [18] and quark [20]. The obtained results indicate that *Lactococcus* bacteria were more prone to cell lysis in quark than in gouda. This was especially interesting considering the fact that quark was stored for a shorter period of time than gouda.

Yeast and molds are typical microorganisms that cause spoilage of gouda and quark [17,43]. The use of the FUR + AgNPs film significantly influenced the number of yeast during cheese storage. It slowed down and inhibited the growth of these microorganisms in gouda (Table 1) and quark (Table 2), respectively. Moreover, an increase in the number of yeasts was observed in both controls. An inhibitory effect of the active film on mold count was also observed; however, only regarding gouda. The number of molds increased (*p* ≤ 0.05) in control but these microorganisms were not detected in gouda wrapped in the FUR + AgNPs film after storage. Similarly, chitosan/polyvinyl alcohol/glycerol coating containing titanium dioxide eliminated mold growth on the surface of Ras cheese [39]. On the contrary, the mold count did not differ (*p* > 0.05) in quark after storage regardless of the packaging used. The number of yeasts and molds steadily increases during aging of gouda at 10 °C [44]. Therefore, their growth in control during storage was expected.

The obtained results clearly showed that the active film used improved the microbiological quality of gouda and quark during storage. This was probably due to the presence of AgNPs, as they are highly toxic to a wide range of microorganisms [13]. Other authors have also shown an extension of the shelf life of other food products through the use of films with AgNPs such as: mini kiwi [7], red grapes [45], and strawberries [46,47].

### 3.3. Organoleptic Quality of Cheese

**Figure 1:**
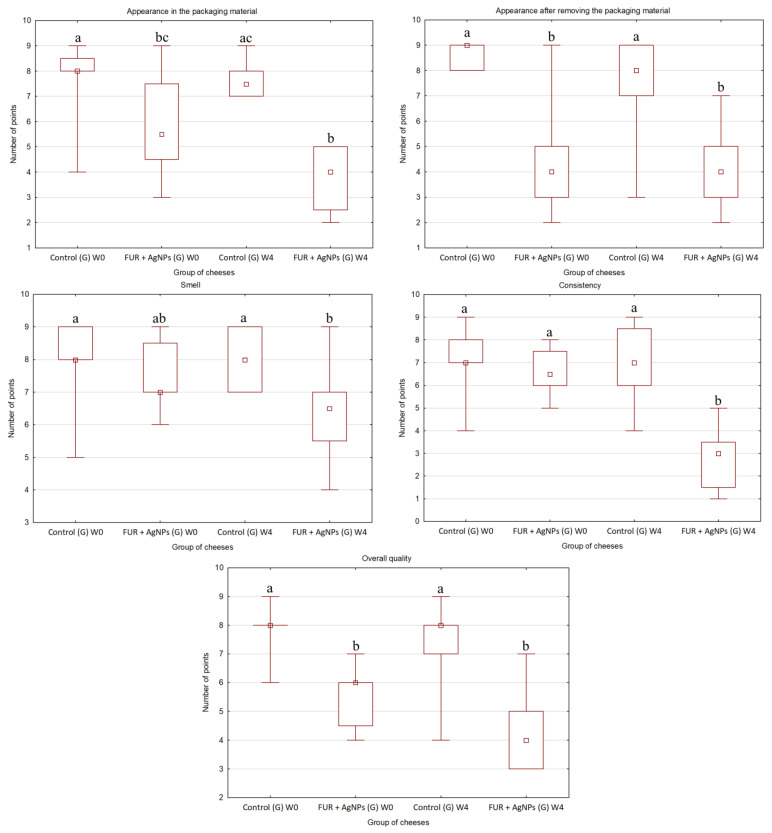
Organoleptic quality of gouda determined in the 9-point hedonic scale before and after storage for 4 weeks at 8 ± 1 °C (different letters mark significant differences (*p* ≤ 0.05) between groups regarding following quality properties). A square represents a median, a rectangle represents quartiles (25–75%), and line segments represent maximum and minimum values. Control: the cheese wrapped in a LLDPE film; FUR + AgNPs: the cheese wrapped in an active film; G: gouda; W: before storage; W4: after 4 weeks of storage.

**Figure 2:**
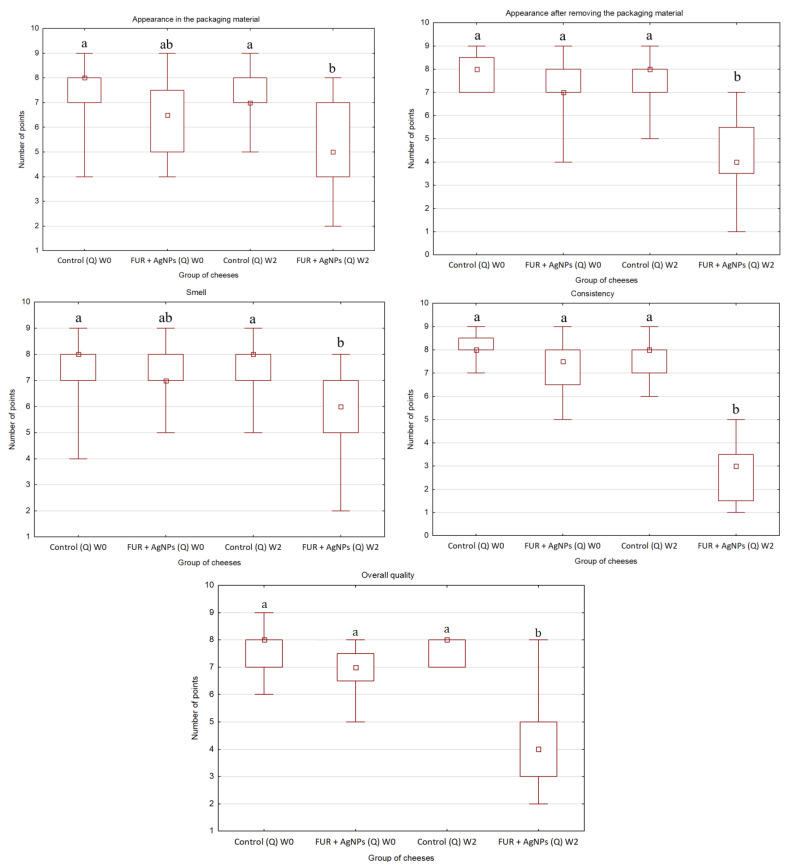
Organoleptic quality of quark determined in the 9-point hedonic scale before and after storage for 2 weeks at 4 ± 1 °C (different letters mark significant differences (*p* ≤ 0.05) between groups regarding following quality properties). A square represents a median, a rectangle represents quartiles (25–75%) and line segments represent maximum and minimum values. Control: the cheese wrapped in a LLDPE film; FUR + AgNPs: the cheese wrapped in an active film; Q: quark; W0: before storage; W2: after 2 weeks of storage.

The results of organoleptic evaluation of gouda and quark at the beginning and after storage are shown in Figure 1 (gouda) and in Figure 2 (quark). Furthermore, the appearance of gouda and quark wrapped in LLDPE and FUR + AgNPs films before and after storage is presented in Table 3.

According to panelists, both the appearance of gouda in the FUR + AgNPs packaging material and after its removal was less desirable (*p* ≤ 0.05) than the control. Panelists had the same impression both at the beginning of storage and at the end of it. The active film was not colorless (Table 3), which was not appreciated by the panelists.

The active packaging material did not affect (*p* > 0.05) the smell and consistency of gouda at the beginning of storage. However, after storage, both these characteristics were assessed by panelists as less desirable (*p* ≤ 0.05) in the cheese wrapped using FUR + AgNPs film than in control. This was probably due to the fact that during storage the smell of cheese changed and became less pronounced and its consistency became harder than the control wrapped in LLDPE. Moreover, the consistency of gouda stored in FUR + AgNPs film was assessed mostly negatively (1–4 points).

The organoleptic evaluation revealed that gouda wrapped in the FUR + AgNPs film was characterized by a worse (*p* ≤ 0.05) overall quality than control regardless of the time of storage.

Unlike gouda cheese, the appearance of quark in the active packaging material and after its removal was found to be less preferable by panelists only after storage. This was observed even though the FUR + AgNPs film was not colorless (Table 3).

The FUR + AgNPs film had the same effect on the smell and consistency of quark as it had on these characteristics regarding gouda. No significant differences were observed between samples of quark at the beginning of storage. Both these features after storage were less desirable (*p* ≤ 0.05) in the cheese wrapped using FUR + AgNPs film than in control. Moreover, the consistency of quark stored in FUR + AgNPs film was assessed mostly negatively (1–4 points). However, unlike gouda, quark packed using the active film showed worse overall quality than control only after storage.

The smell of an acid-curd cheese-like quark usually becomes more intense during storage [22] as does the flavor of gouda [17]. Therefore, the smell of both cheese varieties wrapped in the active film was less appreciated by panelists than that of control. The change in consistency was a consequence of the decrease in water content as a result of a much higher WVTR of the FUR + AgNPs [26] than LLDPE [40] film.

Amjadi et al. [42] also determined a reduction in the hedonic scores of smell and overall acceptance during the storage of white cheese wrapped in films containing zinc oxide nanoparticles. However, the same effect was observed in control.

Flavor, then texture and appearance, are the three organoleptic characteristics that have the greatest impact on the attractiveness of a food product [19]. Consequently, smell, consistency, and appearance of cheese should not be negatively affected by a packaging material. Even though the examined FUR + AgNPs film prolongs the shelf life of cheese, it needs to be improved in order to eliminate the final negative impact on cheese organoleptic quality.

### 3.4. Migration of Silver Nanoparticles

The content of silver in gouda and quark before and after storage is presented in Table 4. Silver was only detected in the samples after storage. In gouda it was present in the outer part of the sample, while in quark it was found both in the rind and in the interior. As expected, more silver was determined in the part of quark which was in direct contact with the FUR + AgNPs film.

The migration of silver nanoparticles into gouda was slower than in the case of the quark. It was probably caused by the differences in the content of water in the investigated kinds of cheese (Table 1 and Table 2). Gouda, mainly due to its lower water content and a different method of coagulation, has a more solid structure than quark. Moreover, the level of silver was below the limit of quantification in the interior of gouda.

According to Simbine et al. [1], silver nanoparticles may be incorporated into non-degradable and biodegradable polymers in order to manufacture food packaging materials. However, there are only a few references that present the results of the migration of silver from packaging into cheese. The amount of silver determined in the interior of quark was similar to the results obtained by Li et al. [48] in refrigerated cottage cheese packed using PLA/TiO_2_ + Ag nanocomposite film (approximately 0.020 mg/kg after 25 days of storage) and by Metak et al. [49] in soft cheese packed using nano-silver coated film (approximately 0.025 mg/L after 10 days of storage). Moreover, Introna et al. [50] also confirmed the silver migration in the fior di latte cheese wrapped in silver nano-particle coating. They did not, however, determine the exact amount.

The migration limit of silver from plastic materials and articles into food or food simulants has not been established. According to Commission Regulation (EU) no. 10/2011 [51], the lowest migration limit of heavy metals was set for nickel (0.02 mg/kg food or food simulant) while the highest was for iron (48 mg/kg food or food simulant). The content of silver in gouda and quark after storage were far below the limit established for iron. According to Cushen et al. [15], a provisional ingestion limit value of silver is 0.482 mg/kg (body weight)/day). Therefore, the investigated cheeses do not seem to pose a threat to human health. Nevertheless, adequate toxicological data of active packaging materials containing AgNPs is not yet available [15].

## 4. Conclusions

The conducted research allowed us to draw the following conclusions:

1. The FUR + AgNPs film applied as packaging of gouda and quark during storage improved the microbiological quality of both cheese varieties;
2. The level of silver detected in the samples after storage seemed to not pose a threat to human health;
3. All organoleptic characteristics of cheeses wrapped in the active film assessed after storage were found to be less desirable than that of control.

Consequently, it is worth continuing research for the improvement of the FUR + AgNPs film in order to enable its use in everyday life.

## Figures and Tables

**Table 1:** The water content, physicochemical properties and microbiological quality of gouda wrapped in LLDPE (control) and FUR + AgNPs (active) films during storage.

Parameters	Type of Film	Storage Weeks	
		0	4
Water content (%)	Control	42.0 ^b^ ± 0.2	41.2 ^b^ ± 1.0
	Active		32.6 ^a^ ± 0.1
Water activity	Control	0.971 ^b^ ± 0.001	0.966 ^ab^ ± 0.002
	Active		0.935 ^a^ ± 0.003
pH	Control	5.64 ^b^ ± 0.01	5.63 ^ab^ ± 0.00
	Active		5.58 ^a^ ± 0.01
*Lactococcus* count (log cfu/g)	Control	9.4 ± 0.3	9.5 ± 0.3
	Active		9.7 ± 0.1
TBC (log cfu/g)	Control	9.8 ± 0.4	10.1 ± 0.1
	Active		9.9 ± 0.2
Yeast count (log cfu/g)	Control	0.6 ^a^ ± 0.5	5.2 ^c^ ± 0.3
	Active		2.6 ^b^ ± 0.3
Mold count (log cfu/g)	Control	0.1 ^a^ ± 0.1	2.5 ^b^ ± 0.2
	Active		ND

Mean values ± standard deviation with different superscript letter are significantly different (*p* ≤ 0.05). ND: not detected.

**Table 2:** The water content, physicochemical properties, and microbiological quality of quark wrapped in LLDPE (control) and FUR + AgNPs (active) films during storage.

Parameters	Type of Film	Storage Weeks	
		0	4
Water content [%]	Control	74.3 ^b^ ± 1.3	75.0 ^b^ ± 0.5
	Active		44.4 ^a^ ± 4.5
Water activity	Control	0.990 ^b^ ± 0.002	0.990 ^b^ ± 0.006
	Active		0.963 ^a^ ± 0.016
pH	Control	4.61 ± 0.02	4.67 ± 0.11
	Active		4.66 ± 0.03
*Lactococcus* count (log cfu/g)	Control	8.3 ^b^ ± 0.1	5.8 ^a^ ± 0.6
	Active		6.3 ^a^ ± 0.7
TBC (log cfu/g)	Control	8.2 ± 0.1	6.2 ± 0.9
	Active		6.0 ± 0.3
Yeast count (log cfu/g)	Control	ND	3.7 ± 1.2
	Active		ND
Mold count (log cfu/g)	Control	ND	0.6 ± 0.5
	Active		1.2 ± 1.0

Mean values ± standard deviation with different superscript letter are significantly different (*p* ≤ 0.05). ND: not detected.

**Table 3:** The appearance of gouda and quark wrapped in LLDPE (control) and FUR + AgNPs (active) films before and after storage.

	Gouda	
Appearance at the beginning of storage	Control 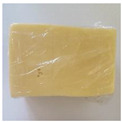	Active 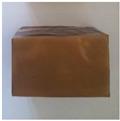
Appearance after four weeks of storage	Control 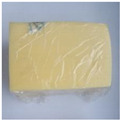	Active 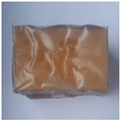
	**Quark**	
Appearance at the beginning of storage	Control 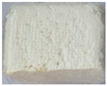	Active 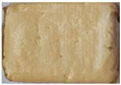
Appearance after two weeks of storage	Control 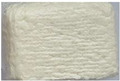	Active 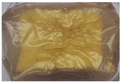

**Table 4:** The content of silver in gouda and quark before and after storage in FUR + AgNPs film.

Type of Cheese	Type of Sample	Content of Silver (mg/kg)
Gouda	Before storage	<LOQ
	After storage (rind)	3.897 ± 1.750
	After storage (interior)	<LOQ
Quark	Before storage	<LOQ
	After storage (rind)	0.717 ^b^ ± 0.690
	After storage (interior)	0.036 ^a^ ± 0.023

LOQ: limit of quantification 0.009 mg/kg. Mean values ± standard deviation with different superscript letters are significantly different (*p* ≤ 0.05).

## Data Availability

Not applicable.
